# Environmental pH modulates inerolysin activity via post-binding blockade

**DOI:** 10.1038/s41598-018-19994-8

**Published:** 2018-01-24

**Authors:** Ryan Rampersaud, Emma L. Lewis, Timothy J. LaRocca, Adam J. Ratner

**Affiliations:** 1 College of Physicians & Surgeons, Columbia University, New York, NY USA; 2 Department of Pediatrics, New York University School of Medicine, New York, NY USA; 3 Department of Microbiology, New York University School of Medicine, New York, NY USA; 4 Present Address: Department of Psychiatry, University of California, San Francisco, CA USA; 5 Present Address: Perelman School of Medicine, University of Pennsylvania, Pennsylvania, PA USA; 6 Present Address: Department of Basic & Clinical Sciences, Albany College of Pharmacy and Health Sciences, Albany, NY USA

## Abstract

The cholesterol dependent cytolysins (CDCs) are a family of pore-forming toxins produced by a wide range of bacteria. Some CDCs are important virulence factors for their cognate organisms, but their activity must be tightly regulated to ensure they operate at appropriate times and within the appropriate subcellular compartments. pH-dependent activity has been described for several CDCs, but the mechanism of such regulation has been studied in depth only for listeriolysin O (LLO), which senses environmental pH through a triad of acidic residues that mediate protein unfolding. Here we present data supporting a distinct mechanism for pH-dependence for inerolysin (INY), the CDC produced by *Lactobacillus iners*. Inerolysin (INY) has an acidic pH optimum with loss of activity at neutral pH. INY pH-dependence is characterized by reversible loss of pore formation with preservation of membrane binding. Fluorescent membrane probe assays indicated that INY insertion into host cell membranes, but not oligomerization, was defective at neutral pH. These data support the existence of a newly appreciated form of CDC pH-dependence functioning at a late stage of pore formation.

## Introduction

Pore-forming toxin (PFT) production by bacterial species is a common feature of pathogenic bacteria and can play prominent roles in colonization, invasion and disease^1^. Transcriptional regulation is a common strategy for coordinating toxin production with environmental conditions^2–4^. Such regulation permits only a delayed response to environmental stimuli, as alteration of protein levels must lag transcriptional changes. In contrast, regulation at the level of protein activity can occur rapidly in the face of changing environments.

A common theme among bacterial toxins is alteration of activity by environmental pH. Within the primary structure of the protein, amino acids with charged side chains can adopt protonated or unprotonated states. Because the charge present on these amino acids can be altered rapidly by pH and because toxin activity is dependent on the secondary and tertiary structure of the protein, changes in environmental pH may influence toxin activity. Studies have demonstrated that a number of toxins that are trafficked through the endosomal compartment become partially unfolded, exposing hydrophobic residues and facilitating insertion into membranes, including diphtheria toxin^5^ as well as *Clostridium difficile* toxin B^6^. This feature has also been noted for anthrax toxin, in which low pH induces unfolding of lethal factor (LF) and edema factor (EF). This partial unfolding event is required for the passage of these two factors through the heptameric pore formed by protective antigen (PA)^7^. These rapid alterations of secondary/tertiary structure are critical for proper activity of these various toxins.

The cholesterol dependent cytolysins (CDCs) are a family of protein toxins produced by a wide range of Gram-positive (and a few Gram-negative) organisms^8,9^. CDCs share several characteristics, including a four-domain structure, a requirement for membrane cholesterol for efficient activity, and an ability to form large pores in host cells^10^. In general, soluble CDC monomers are secreted into the extracellular environment and bind to target cell membranes through direct recognition of cholesterol via a Thr-Leu pair^11^. Upon membrane binding, a complex and concerted sequence of events occurs, resulting in CDC homo-oligomerization and subsequent pore formation.

While much is known about CDC structure and pore formation, regulation of CDC expression and activity are less well understood. Regulation of toxin expression has been shown for anthrolysin O (ALO), whereby both oxygen status and glucose levels affect production by *B. anthracis*^2^. With regard to regulation at the level of activity, listerolysin O (LLO) from *Listeria monocytogenes* is perhaps the most studied CDC, showing an acidic pH optimum, with little or no activity at neutral pH due to protein unfolding^8^. Further studies demonstrated that this acidic pH optimum and mechanism of regulation was common to *Listeria* derived CDCs^12^. Previous work from our group and others has demonstrated that pH-regulated activity is a feature of a number of CDCs, including perfringolysin O (PFO) from *Clostridium perfringens*, vaginolysin (VLY) from *Gardnerella vaginalis*, intermedilysin (ILY) from *Streptococcus intermedius*, and INY from *Lactobacillus iners*^13–15^. Our previous study of INY suggested that the molecular basis of its acidic pH optimum was distinct from that of the *Listeria* derived toxins^14^, but the mechanisms of CDC pH-dependence among the non-*Listeria* CDCs remain uncharacterized. Here we show that the mechanism for pH dependence in INY involves blockade at a stage that follows membrane binding and toxin oligomerization.

## Results

### The pH dependence of CDC activity is not due to protein degradation

**Figure 1:**
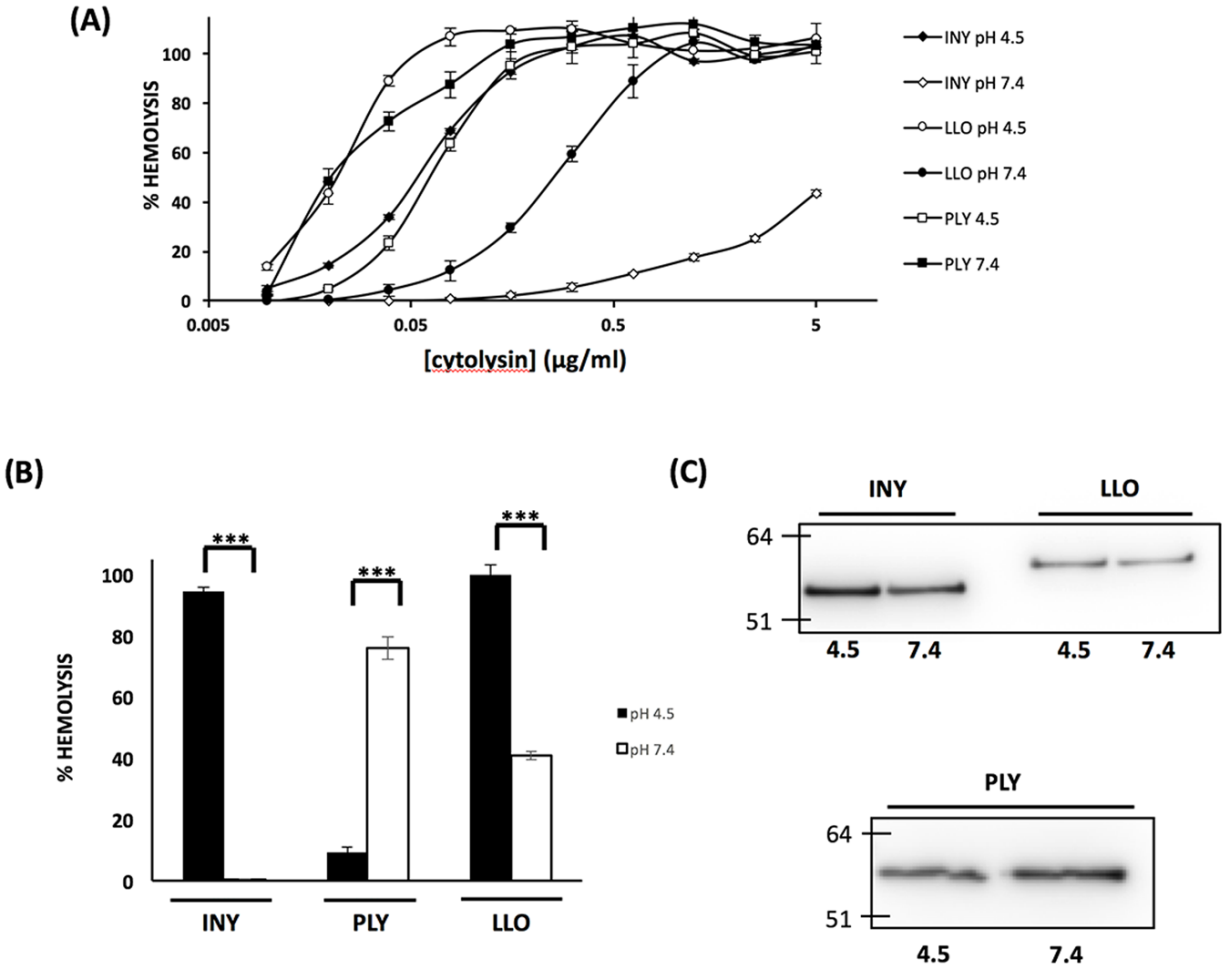
INY has pH dependent activity that is not due to protein degradation. (**A**) Various concentrations of INY, LLO, and PLY were incubated at pH 4.5 or pH 7.4 at 37 °C for 20 min and used in an endpoint hemolysis assay (**B**) INY, PLY, and LLO (125 ng/mL) were incubated at 37 °C for 20 min and used in an endpoint hemolysis assay. (**C**) Toxin was treated at the indicated pH at 37 °C for 20 minutes. Intact protein was detected with an anti-His tag antibody via western blot.

The CDCs have varying pH dependent profiles. Work from other groups has demonstrated that LLO and other *Listeria* derived cytolysins have acidic pH optima^12^, consistent with their role in escape from the phagosome^16^. PFO also displays an acidic pH optimum, and it has been suggested that this feature is consistent with an intracellular component to its life cycle^17^. Our previous work expanded the repertoire of pH regulated toxins to include INY, VLY, and ILY^14^. Notably, VLY, and ILY showed neutral pH optima, whereas INY demonstrated an acidic pH optimum. Here, INY, pneumolysin (PLY) and LLO recombinant proteins were purified and hemolytic activity assessed as a function of pH (Fig. 1A,B). Consistent with prior findings, INY showed maximum activity at pH 4.5. PLY had the opposite pH dependent profile, with a neutral pH optimum and a significant loss of activity at acidic pH. This finding is in contrast to previously published work that suggested that PLY was “pH insensitive”; however, in that work PLY activity was only assessed at pH ≥ 6.0^12^. Prior work examining the molecular basis of LLO activity has suggested that loss of activity is a result of conformational change rather than alterations in protein abundance^8^. Protein levels were assessed by western blot analysis, after treatment at the indicated pH for 20 min at 37 °C (Fig. 1C). LLO, INY, and PLY all demonstrate no loss of protein despite loss of activity.

### INY exhibits reversible loss of activity at neutral pH

**Figure 2:**
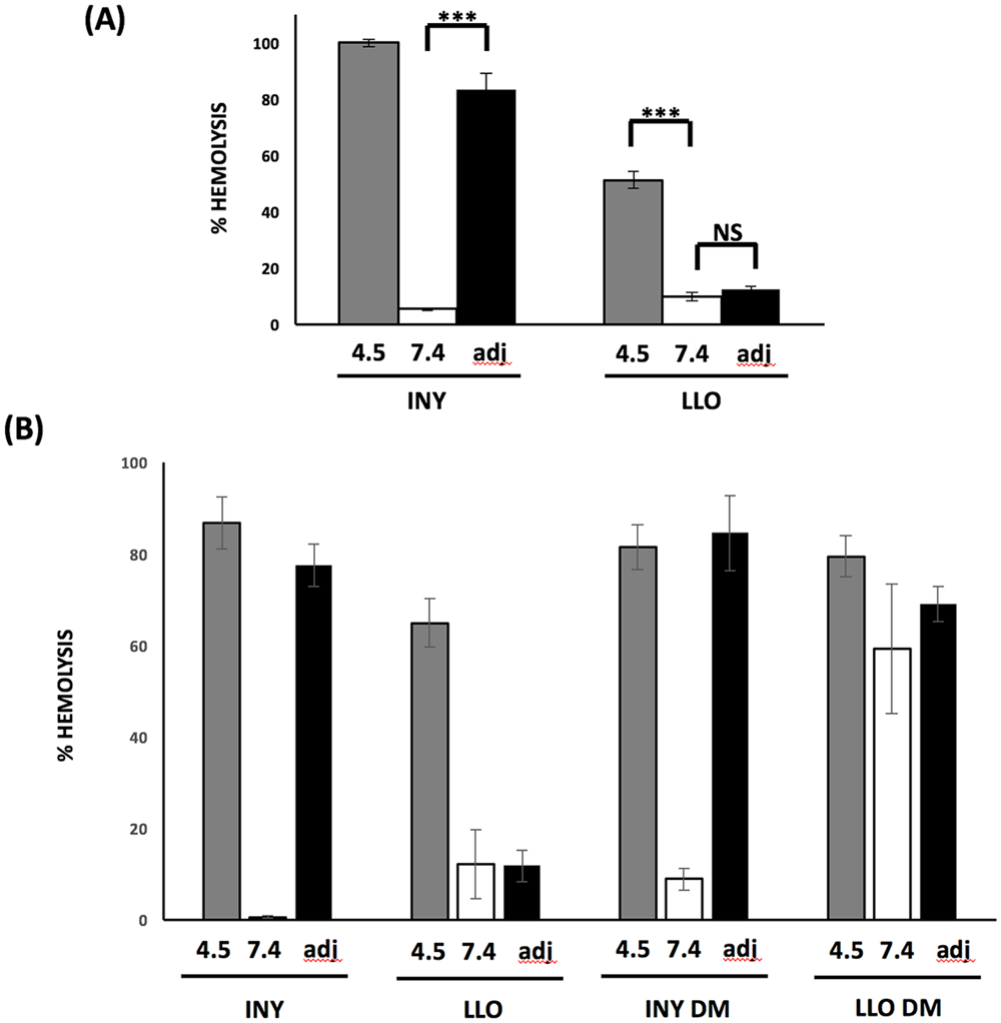
INY has reversible pH dependent activity. INY was incubated at either pH 4.5 or pH 7.4 for 20 minutes at 37 °C followed by incubation at 4 °C for an additional 20 min before use in an endpoint hemolysis assay. For the adjusted sample (adj), INY was incubated at pH 7.4 and the pH of these samples was adjusted to pH 4.5 and incubated for an additional 20 min at 4 °C before use in a hemolysis assay (**B**) Residues in INY were mutated to resemble LLO (INY DM) and residues in LLO were mutated to resemble INY (LLO DM) and incubated at either pH 4.5 or 7.4 for 20 minutes at 37 °C followed by incubation at 4 °C for an additional 20 min before use in an endpoint hemolysis assay. Adjusted samples (adj) were treated as before prior to use in hemolysis assay.

The structural basis for LLO pH dependence has been investigated. Upon exposure to neutral pH, a rapid and irreversible unfolding of domain 3 occurs, which results in a loss of activity^8^. The molecular basis for this activity has been localized to a triad of acidic residues (Glu-247, Asp-320, Asp-208) present in domain 3 of the protein^8^. However, other work has suggested that the LLO acidic pH optimum depends on a unique histidine residue that controls pore conductance^18,19^. That histidine is not present in the INY amino acid sequence. We confirmed the irreversible nature of LLO inactivation at neutral pH using an adjustment strategy (i.e. treating the toxin at pH 4.5 and 37 °C or pH 7.4 and 37 °C, followed by adjusting the pH to 4.5 prior to testing activity; (Fig. 2A). In contrast to LLO, INY, which lost activity at pH 7.4, regained activity by shifting the pH to 4.5 (Fig. 2A). These results suggest that the pH dependent activity of INY is distinct from that of LLO, given its reversible nature. We next determined if the analagous residues in INY (Ser-239, Asp-312, Thr-200) were responsible for the pH dependent activity of INY. An INY mutant was generated in which Ser-239 was changed to a glutamate and Thr-200 was changed to an aspartate, to create an “LLO-like” mutant version of INY (INY DM), and subsequently used this mutant to assess activity at pH 4.5, pH 7.4, and after pH adjustment (as before). The INY DM had pH dependent activity similar to native INY, showing a significant reduction in activity after incubation at pH 7.4 but recovery on return to pH 4.5 (Fig. 2B**)**. We also generated an “INY-like” mutant version of LLO (LLO DM) by changing Glu-247 to serine and Asp-208 to threonine and assessed activity at pH 4.5, pH 7.4, and after pH adjustment. This mutant protein lost pH dependent activity, showing no difference in hemolysis between pH treatments, consistent with loss of the acidic sensor residues. These results suggest that pH dependent activity in INY is localized to an area of the protein distinct from that in LLO.

### Membrane binding activity of pH-treated CDCs remains intact

**Figure 3:**
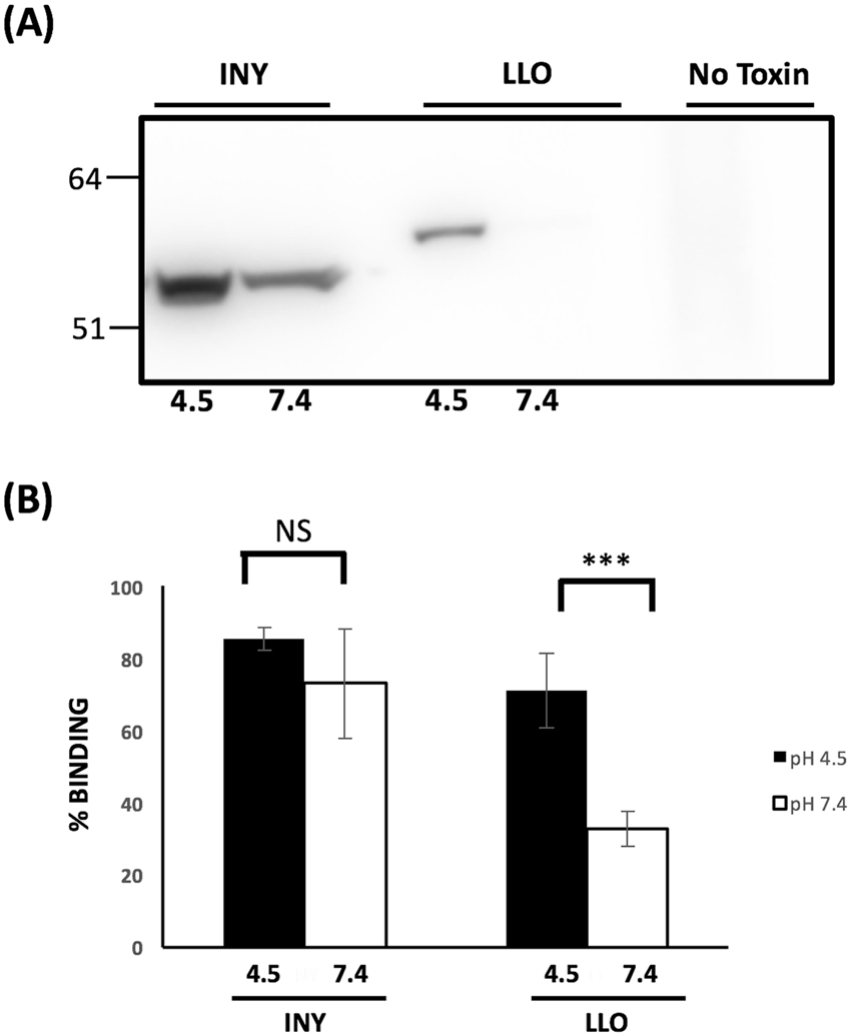
Membrane binding activity of INY remains intact at neutral and acidic pH. (**A**) INY and LLO (100 ng/mL) were incubated with human RBCs for 5 min on ice and then membrane binding was assessed by western blot (**B**) Cholesterol (2 mg/well) was immobilized on a PVDF membrane. INY was treated at pH 4.5 (black bars) or 7.4 (white bars) and incubated for 1 h. INY binding was analyzed by ELISA.

Pore formation in the CDCs occurs in a concerted and stereotyped manner. The first step involved in pore formation is the association of soluble monomer with its cellular receptor (cholesterol for most CDCs; hCD59 for VLY, ILY, and lectinolysin)^10,20,21^. Briefly, INY was treated at the indicated pH, incubated with RBCs at 4 °C, unbound toxin was washed away, and bound toxin was detected using an anti-His antibody. At pH 7.4, a condition in which hemolytic activity of INY is almost completely lost, RBC binding activity remained present (Fig. 3A). In contrast, LLO binding was lost under analogous conditions. (Fig. 3A). A majority of the CDCs utilize cholesterol as their cell surface receptor mediating recognition with target cells. In order to further address this step of pore formation, we examined whether direct binding to cholesterol was impaired after pH treatment. INY was treated at either pH 4.5 or pH 7.4 and added onto PVDF coated with cholesterol. Binding was quantified by ELISA using an anti-His antibody. INY showed no significant difference in cholesterol binding capacity with either pH treatment (Fig. 3B). In contrast, LLO, which unfolds at neutral pH, had significantly reduced cholesterol binding activity.

### INY shows no defect in oligomerization at neutral pH

**Figure 4:**
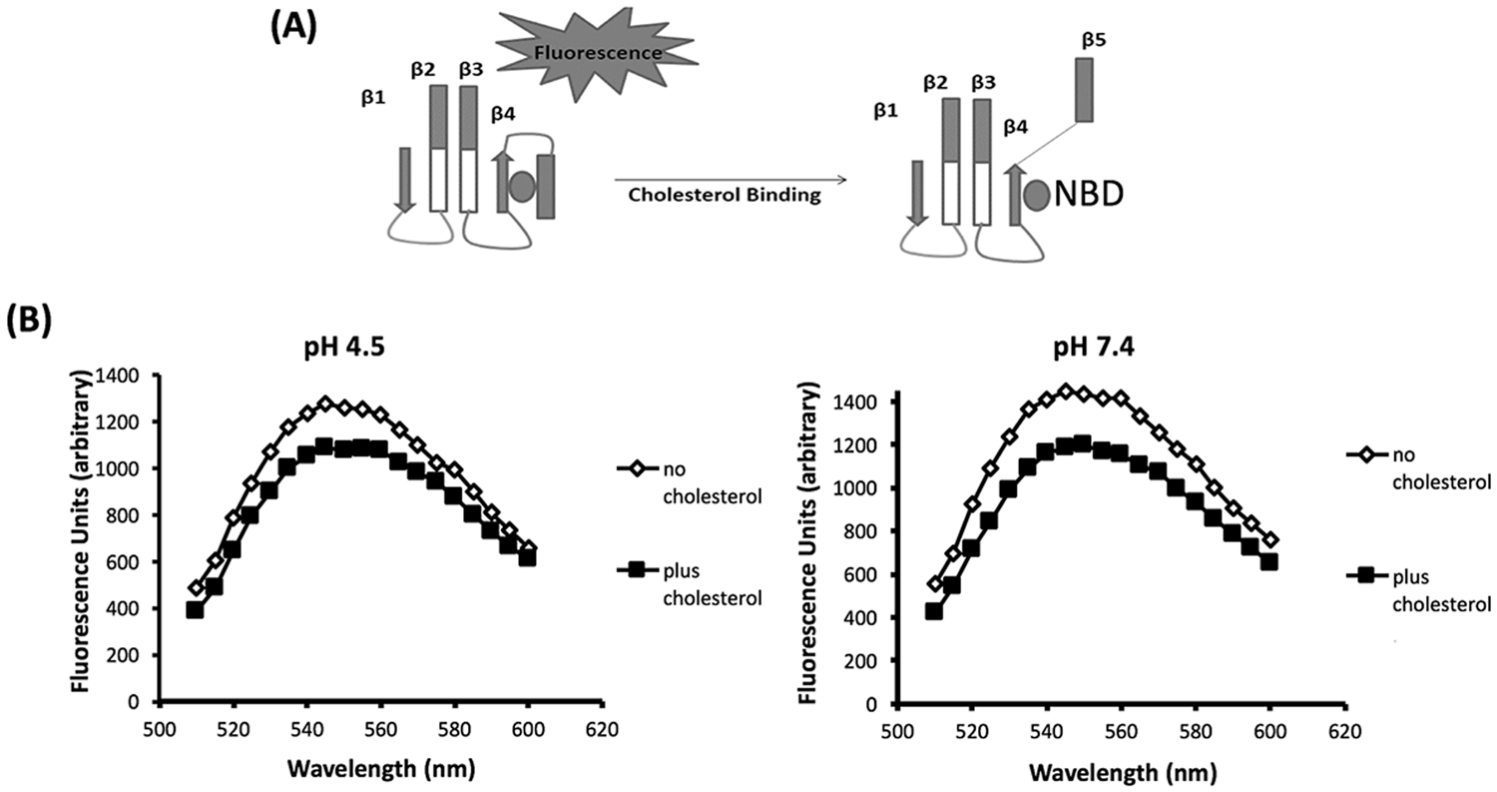
Fluorescent detection of INY oligomerization at acidic and neutral pH. (**A**) Schematic of fluorescent-based oligomerization assay. During oligomerization, conformational change takes place that causes a transition of the NBD dye from a nonpolar to a polar environment and a subsequent reduction in fluorescence intensity. (**B**) INY-I339C was labeled with NBD and fluorescence emission measured in the presence or absence of cholesterol after a 30 min incubation at 37 °C at pH 4.5 (left) or pH 7.4 (right).

Upon membrane binding of soluble monomers to cholesterol via the Thr-Leu pair, loops L2 and L3 insert into the membrane, as does the undecapeptide region^11^. Specifically, upon insertion of the undecapeptide changes are transmitted to domain 3 of the protein, and specifically to the four β sheets that contribute to the final pore. The β4 sheet is normally not exposed due to hydrogen bonding to a short hydrophobic region termed the β5 loop. Upon membrane binding, insertion of a tryptophan in domain 3 into the membrane initiates the movement of the β5 loop away from the β4 sheet by virtue of a flexible glycine linker. This exposes β4, which then allows it to interact with the exposed surface of the β1 sheet allowing for oligomerization into a prepore complex. To assess competence for oligomerization, we monitored specific structural transitions that mediate this process. Upon membrane binding, the rotation of β5 loop away from the β4 sheet contributes to the formation of an SDS-resistant prepore, and this can be monitored spectroscopically using an environmentally sensitive probe conjugated to a cysteine residue substituted for Ile-339 in β4^22^. Initially buried under the β5 sheet and in a nonpolar environment, this residue becomes exposed to the polar environment and fluorescence is quenched. Figure 4A is a schematic of these events. At either pH, we observed a comparable reduction in fluorescence intensity, indicating the presence of these structural changes in both pH treated toxins (Fig. 4B). Taken together, our results suggest that at either pH, despite a loss of hemolytic activity at pH 7.4, INY was competent for oligomerization. It is important to note that the location of the probe in these experiments allows assessment of only oligomerization and not subsequent steps in pore insertion.

### CDCs have a defect in membrane insertion after pH treatment

**Figure 5:**
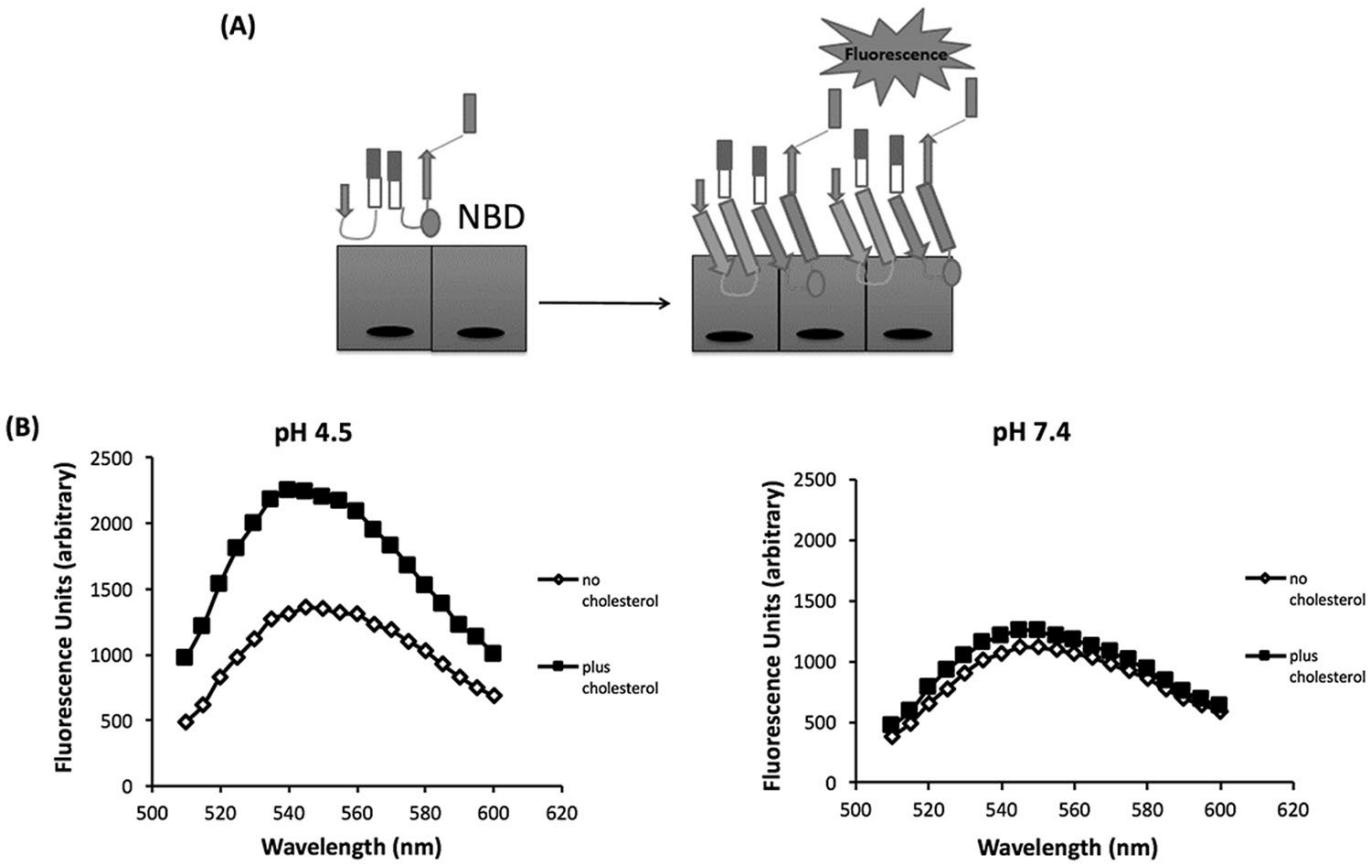
Defective INY membrane insertion at neutral pH. (**A**) Schematic of events in fluorescence assay. INY-D305C was labeled with NBD and incubated with cholesterol. After oligomerization and upon final pore formation the labeled residue inserts into the membrane and undergoes a transition from a polar to a nonpolar environment and a resultant increase in fluorescence intensity. (**B**) Fluorescence emission was measured in the presence or absence of cholesterol after a 30 min incubation at 37 °C at pH 4.5 (left) or pH 7.4 (right).

Having determined that at both acidic and neutral pH, membrane binding activity was intact and INY was competent for oligomerization, we assessed the final step in pore formation, insertion of the transmembrane helices (TMH) into the target cell membrane. In order to monitor these structural changes, we conjugated the environmentally sensitive probe NBD to a cysteine residue substituted for Asp-305 in TMH2. Figure 5A is a schematic of the events in the final step of pore formation. Upon incubation with cholesterol, we found that at pH 4.5 there was a significant increase in fluorescence intensity (Fig. 5B). In contrast, at pH 7.4, toxin incubated with cholesterol showed no increase in fluorescence intensity, indicating a lack of insertion of transmembrane helices at this elevated pH (Fig. 5B). In combination with the results from the oligomerization probe studies, these findings were consistent with a late-stage blockade of INY activity, preventing membrane insertion, at pH 7.4.

## Discussion

While much is understood about the structure and function of CDCs, less is known about its regulation. The use of pH as a regulatory factor is common among proteins^7,23^, allowing for rapid regulation of their activity via conformational changes. pH dependent activity has been identified for a number of CDCs, including ILY, VLY, INY, PFO, and LLO^8,13,14^. Our prior work demonstrated that the basis of pH dependence in INY was likely distinct from the unfolding-based mechanism observed in LLO. In this work, we investigated the mechanism of pH dependent activity of INY and demonstrated that loss of function occurs at the final stages of pore formation. The loss of activity seen with INY likely represents a reversible conformational change of the protein (and not a global unfolding) given our findings that activity can be restored by a return to acidic pH.

Our work demonstrates that INY is competent for membrane binding and suggests that oligomerization, the rate limiting step of pore formation, is intact at pH 7.4^24^. Rather, it is the last step of the pore forming mechanism, the insertion of the transmembrane domains, which is impaired. The final steps of pore formation have not yet been fully elucidated, but previous work suggests that monomer-monomer interactions drive a conversion of the alpha helices in TMH1/2 into β sheets, followed by a concerted insertion of these regions to form the final functional pore structure. Interactions between toxin monomers are required for pore formation^25^, and the data presented here are consistent with the recent characterization of intramolecular disulfide bonds as crucial mediators of prepore-to-pore conversion in CDCs^26^.

These studies demonstrate that for INY, pH dependent activity operates through a mechanism distinct from that identified for LLO. At neutral pH, INY has a defect that occurs after the initial membrane binding step and oligomerization. Our results using fluorescent probes to monitor structural changes, suggest that membrane insertion is defective at neutral pH. These results highlight a novel form of regulation for INY and suggest that the pH dependent activity of LLO may not be conserved across CDCs. These two distantly related toxins seemed to have evolved acidic pH optima independently, consistent with their anatomical niches. *Lactobacillus iners* colonizes the human vagina^27^, and INY is suited to operating within that normally acidified niche. Given that a number of other closely related CDCs (ILY, VLY, and PLY) also show pH dependent activity, we speculate that these toxins may have similar mechanisms. Future studies will be aimed at determining whether the late-stage blockade is present in other CDCs, especially those whose cognate organisms inhabit a similar niche to that of *L. iners*.

## Methods

### Production of recombinant CDCs

Cloning, expression, and purification of recombinant CDCs has been previously described^14^. Site-directed mutagenesis was carried out using overlap-extension PCR using the indicated primers and confirmed by Sanger sequencing. Constructs were cloned into pET28a using *NdeI* and *XhoI* sites, and confirmed by sequencing. Protein purification was carried out as previously described^14^. The listeriolysin O (LLO) ORF lacking its predicted signal sequence was amplified by PCR from *L. monocytogenes* BAA751 genomic DNA using primers BamHI-LLO-F (GCCGCCGGATCCAAGGATGCATCTGCATTCAATAAAG) and LLO-R-XhoI (GCCGCCCTCGAGTTATTCGATTGGATTATCTAC). For the creation of the INY double mutant, Thr-200 was changed to a glutamate using primer pair primers NheI-INY-F (GCCGCCGCTAGCAATACTGAGCCAAAAACAGCTATTG) and INY-Thr200-GluR(GCTTTGAGCACTTTCTGTATCATATTCAATTC) and INY-Thr200-GluR(GAATTGAATATGATACAGAAAGTGCTCAAAGC) and XhoI-INY-R (GCCGCCCTCG AGTTAGTCATTTTTTACTTCTTCTTTG). This was followed by replacement of a Ser-239 with a glutamate using NheI-INY-F (GCCGCCGCTAGCA ATACTGAGCCAAAAACAGCTATTG) and INY-Ser239-GluR (GCTTAAAGTTAACAATCTCTTTCTGCTTGTTTCTC) and INY-Ser239-GluF (GAGAAACAAGCAGAAAGAGATTGTTAACTTTAAGC) and XhoI-INY-R (GCCGCCCTCG AGTTAGTCATTTTTTACTTCTTCTTTG). In order to construct the LLO double mutant, Asp-208 was changed to a threonine using primer pairs BamHI-LLO-F (GCCGCCGGATCCAAGGATGCATCTGCATTC AATAAAG) and LLOD208T-R (CACTGTAAGCCATTTCAGTATCATAATCAATTTTTGC) and LLOD208T-F (GCAAAAATTGATTATGATACTGAAATGGCTTACAGTG) and LLO-R-XhoI (GCCGCCCTCGAGTTATTCGATTGGATT ATCTAC). This was followed by replacement of Glu-247 with a serine residue using the primer pairs BamHI-LLO-F (GCCGCCGGATCCAAGGATGCATCTGCATTC AATAAAG) and LLOE247S-R(GTTTAAAACTAATGACACTTTCTTGCATTTTCCC) and LLOE247S-F (GGGAAAATGCAAGAAAGTGTCATTAGTTTTAAAC) and LLO-R-XhoI (GCCGCCCTCGAGTTATTCGATTGGATTATCTAC).

### Erythrocyte lysis assay

The use of human erythrocytes from healthy adult volunteers following informed consent was approved by the Columbia University Institutional Review Board and was carried out in accordance with the standards of the Helsinki Declaration. Preparation of erythrocytes was carried out as previously described^14^. Hemolysis assays were carried out in the presence of buffer C (35 mM sodium phosphate, 125 mM sodium chloride) as previously described^8^. Toxins were preincubated at the indicated pH for 30 min before use in an endpoint assay. In the indicated experiments, toxin was treated for 30 min at the indicated pH and then adjusted using HCl or NaOH and incubated for an additional 30 min at 4 °C before use in an endpoint assay. To measure hemolysis, supernatant was removed, and the optical density at 415 nm (OD_415_) was measured.

### Binding activity of recombinant CDCs to membranes

Recombinant CDCs (5 µg/mL) were incubated for 30 min at 37 °C and pH 4.5 or pH 7.4. 400 µl of toxin was mixed with 400 µl of 1% hRBCs on ice for 5 min in Buffer C (35 mM sodium phosphate, 125 mM sodium chloride) at the indicated pH. Cells were recovered by centrifugation (800 *g*) for 10 min at 4 °C. Cells were washed twice with ice cold PBS, and resuspended in 2X SDS buffer + 1% Triton X-100. Bound CDC was detected using an anti-His-tag antibody (Sigma).

### Binding of recombinant CDCs to immobilized cholesterol

Binding of CDCs to cholesterol was assessed by a PVDF binding assay^12^. Briefly, 20 mg/mL cholesterol (dissolved in 1:1 chloroform:ethanol) was diluted to 20 μg/mL (dissolved in 1:5 chloroform:ethanol), and 100 μl was added to each well (2 μg per well) of a 96 well plate with Immobilon-P membrane (Millipore) at the bottom and allowed to dry overnight. The cholesterol-coated wells were treated with blocking buffer consisting of 4% heat-inactivated fetal bovine serum in PBS for 1 h. CDCs (2 µg/mL) were treated for 30 min at either 37 °C or 4 °C in Buffer C (35 mM sodium phosphate, 125 mM sodium chloride) at pH 4.5 or pH 7.4. Toxin was diluted to 2 nM in Buffer C with 4% heat inactivated fetal bovine serum and 100 µl was added to each well. After 2 hours, wells were washed four times with blocking buffer, and treated with anti His-tag antibody (1:2000) for 1 hour. Wells were washed four times with blocking buffer and antibody was detected with horseradish peroxidase-conjugated anti-mouse IgG at a dilution of 1:2000. The binding of CDCs to cholesterol was determined quantitatively by the addition of 100 μl of 3,3β,5,5β-tetramethylbenzidine (TMB). The reaction was stopped by the addition of 50 µl of 2N H_2_SO_4_ and absorbance was measured at 450 nm. For INY and LLO, percent binding was measured relative to toxin treated at pH 4.5 and 4 °C.

### Protein degradation assay

CDCs (5 µg/mL) were treated for 37 °C in buffer C (35 mM sodium phosphate, 125 mM sodium chloride) at pH 4.5 or 7.4 for 30 min. Proteins were detected by western blot analysis using anti-His-tag antibody. Primary antibody was detected using horseradish peroxidase-conjugated anti-mouse IgG at a dilution of 1:1000 and detected by enhanced chemiluminesence (Roche).

### Construction of INY derivatives and chemical modification of cysteine residues with IANBD

For creation of the NBD labelled mutants, mutation of Cys-476 to alanine using primer pair NheI-INY-F (GCCGCCGCTAGCAATACTGAGCCAAAAACAGCTATTG) and INY-CysAlaF (GAATGTTAAGATTCAAGAAGCTACAGGCTTGGCATG) and primer pair INY-CysAla-R (CATGCCAAGCCTGTAGCTTCTTGAATCTTAACATTCA) and XhoI-INY-R (GCCGCCCTCGAGTTAGTCATTTTTTACTTCTTCTTTG). These two products were then joined together using an overlap extension PCR (OEPCR) strategy. The cysteine-deficient mutant was then used as a template to generate a substitution of Asp-305 to cysteine as well as a substitution of Ile-339 to cysteine for labelling with NBD. Asp-305 was substituted using the primer pairs NheI-INY-F and INY-AspCys-F (GGAAACAACAAGCAGAGTTGCAAAGTTCAAGCAGCTTTTG) and primer pair XhoI-INY-R and INY-AspCys-R (CAAAAGCTGCTTGAACTTTGCAACTCTTGCTTGTTGTTTCC). The two products were then joined in a final OEPCR. The same strategy was undertaken for the isoleucine to cysteine mutants using the primers INY-IleCys-F (ACTAGTGTTGTAGCTGTTTGCTTAGGTGGTAACTC) and INY-IleCysR (GAGTTACCACCTAAGCAAACAGCTACAACACTAGT). Proteins were purified as indicated previously. The indicated toxins were treated with 2.5 mM DTT for 10 minutes at room temperature and then dialyzed overnight to remove DTT. Toxin derivatives were labeled with a 20-fold molar excess of IANBD [iodoacetamido-N,N′-dimethyl-N-(7 nitrobenz-2-oxa-1,3-diazolyl)ethylene-diamine; Molecular Probes] overnight at 4 °C. Following modification with the probe, excess was quenched with 1 mM DTT for 10 min, and probe was removed via extensive dialysis. NBD measurements were carried out with an Infinite 200 microplate reader (Tecan) using the following settings: an excitation wavelength of 480 nm and an emission wavelength of 540 nm with a bandpass of 5 nm. Emission intensity was scanned between 500 and 600 nm at a resolution of 1 nm with an integration time of 1 sec. 10 µg of toxin was incubated with varying amounts of cholesterol in Buffer C for 30 minutes at 37 °C. The fluorescence intensity of the unlabeled samples was subtracted from that of the fluorescent probe-labeled samples in order to control for the intrinsic fluorescence of the sample in the absence of probe.

### Statistical methods

Hemolysis assay results are expressed as average values from three readings from one representative experiment. Each experiment was repeated three times. Data were analyzed by Student t test. A P value of < 0.05 was considered significant.

### Ethics

The use of human erythrocytes following informed consent was approved by the Columbia University Institutional Review Board and carried out in accordance with the standards of the Helsinki Declaration.

### Data Availability

All data generated or analysed during this study are included in this published article.
